# *CCDC102B* confers risk of low vision and blindness in high myopia

**DOI:** 10.1038/s41467-018-03649-3

**Published:** 2018-05-03

**Authors:** Yoshikatsu Hosoda, Munemitsu Yoshikawa, Masahiro Miyake, Yasuharu Tabara, Noriaki Shimada, Wanting Zhao, Akio Oishi, Hideo Nakanishi, Masayuki Hata, Tadamichi Akagi, Sotaro Ooto, Natsuko Nagaoka, Yuxin Fang, Nagahama Study group, Takahisa Kawaguchi, Kazuya Setoh, Yoshimitsu Takahashi, Shinji Kosugi, Takeo Nakayama, Kyoko Ohno-Matsui, Ching-Yu Cheng, Seang Mei Saw, Ryo Yamada, Fumihiko Matsuda, Akitaka Tsujikawa, Kenji Yamashiro

**Affiliations:** 1 Department of Ophthalmology and Visual Sciences, Kyoto University Graduate School of Medicine, Kyoto, 6068507 Japan; 2 Center for Genomic Medicine, Kyoto University Graduate School of Medicine, Kyoto, 6068503 Japan; 3 Department of Ophthalmology and Visual Science, Tokyo Medical and Dental University, Tokyo, 1138510 Japan; 4 Singapore Eye Research Institute, Singapore National Eye Centre, Singapore, 168751 Singapore; 5 Ophthalmology and Visual Sciences Academic Clinical Program, Duke-NUS Medical School, Singapore, 169857 Singapore; 6 Department of Ophthalmology, Yong Loo Lin School of Medicine, National University of Singapore, Singapore, 199228 Singapore; 7 Saw Swee Hock School of Public Health, National University of Singapore, Singapore, 117549 Singapore; 8 Department of Ophthalmology, Otsu Red-Cross Hospital, Otsu, 5208511 Japan; 9 Department of Health Informatics, Kyoto University School of Public Health, Kyoto, 6068501 Japan; 10 Department of Medical Ethics, Kyoto University School of Public Health, Kyoto, 6068501 Japan

## Abstract

The incidence of high myopia is increasing worldwide with myopic maculopathy, a complication of myopia, often progressing to blindness. Our two-stage genome-wide association study of myopic maculopathy identifies a susceptibility locus at rs11873439 in an intron of *CCDC102B* (*P* = 1.77 × 10^−12^ and *P*_corr_ = 1.61 × 10^−10^). In contrast, this SNP is not significantly associated with myopia itself. The association between rs11873439 and myopic maculopathy is further confirmed in 2317 highly myopic patients (*P* = 2.40 × 10^−6^ and *P*_corr_ = 1.72 × 10^−4^). *CCDC102B* is strongly expressed in the retinal pigment epithelium and choroids, where atrophic changes initially occur in myopic maculopathy. The development of myopic maculopathy thus likely exhibits a unique background apart from the development of myopia itself; elucidation of the roles of *CCDC102B* in myopic maculopathy development may thus provide insights into preventive methods for blindness in patients with high myopia.

## Abstract (web summary)

Myopic maculopathy is a complication of myopia that often progresses to blindness. Here, in a genome-wide association study, Hosoda et al. find that rs11873439 intronic to *CCDC102B* is associated with myopic maculopathy, but not with myopia, thus representing a risk factor independent of myopia.

## Introduction

The incidence of myopia is rapidly increasing worldwide^1–3^. Although patients with myopia do not usually suffer from visual disturbance with the aid of glasses or contact lenses, when the degree of myopia becomes severe and worsens to the stage of “high myopia”, complications can occur, leading to reduced vision or blindness. The main vision-threatening complication in high myopia is myopic maculopathy, in which the retinal pigment epithelium (RPE) and choroid beneath the neural retina become severely atrophic, resulting in atrophy of the whole retina and choroid at the macula, the center part of the posterior pole in the eyeball. Myopic maculopathy is a progressive disease that constitutes the leading cause of blindness in Japanese individuals^4^ and the second-leading cause of low vision in Chinese individuals^5,6^. Even though myopia is less prevalent in Caucasians, myopic maculopathy is also a significant cause of blindness in Caucasians^7–11^.

Eyeball enlargement in highly myopic eyes, particularly axial length elongation, significantly contributes to the development of myopic maculopathy^12,13^. However, not all eyes with longer axial length develop myopic maculopathy, and it cannot be predicted which eyes will develop myopic maculopathy and blindness. In addition, eyes without high myopia can also develop fundus changes associated with myopic maculopathy. Discovery of the unique background for developing myopic maculopathy distinct from the background of developing myopia may lead to the establishment of novel preventive methods for myopic maculopathy and blindness. Conversely, if myopic maculopathy constitutes an unavoidable consequence of myopia progression, blindness in high myopia could not be prevented by targeting myopic maculopathy.

Recent genetic studies have revealed genes associated with the development of myopia and high myopia^14,15^. As high myopia is predominant in East Asia, genetic studies on high myopia have been conducted mainly in this region^16–23^. However, it remains unclear whether genetic background affects the development of vision-threatening complications in highly myopic eyes.

Myopic maculopathy is classified into five grades according to severity; categories 2, 3, and 4 are regarded as the pathological state, whereas categories 0 and 1 are considered the physiological state^24^. In the present study, we aimed to identify genes associated with vision-threatening complications in high myopia by performing a two-stage genome-wide association study (GWAS) using myopic maculopathy grade in a Japanese community-based cohort that included participants with all refractive states; i.e., hyperopia, emmetropia, low-grade myopia, and high myopia. Furthermore, we evaluated associations between the discovered gene and myopic maculopathy grade using only highly myopic eyes to confirm that the identified gene contributed to the development of myopic maculopathy and subsequent low vision and blindness in highly myopic eyes. Our findings provide important insights into susceptibility genes for the development of myopic maculopathy distinct from myopia progression.

## Results

### Two-stage GWAS for myopic maculopathy

The Nagahama cohort demographics used in this study are shown in Supplementary Table 1. Myopic maculopathy was graded in all fundus photographs of the participants, and participants with category 0 or 1 in both eyes were used as controls, whereas participants with category 2, 3, or 4 in at least one eye were used as cases of myopic maculopathy. We compared genotype distributions between myopic maculopathy cases and controls using logistic regression analysis. Age, sex, and axial length were used as covariates for adjustment. The axial length of the eye with the more severe grade was chosen, and the longer axial length was used when the grade was the same in both eyes. Although we did not include principal components in covariates for the adjustment, an inflation factor (λGC) of 1.025 indicated good control of population substructure (Supplementary Fig. 1).

**Fig. 1:**
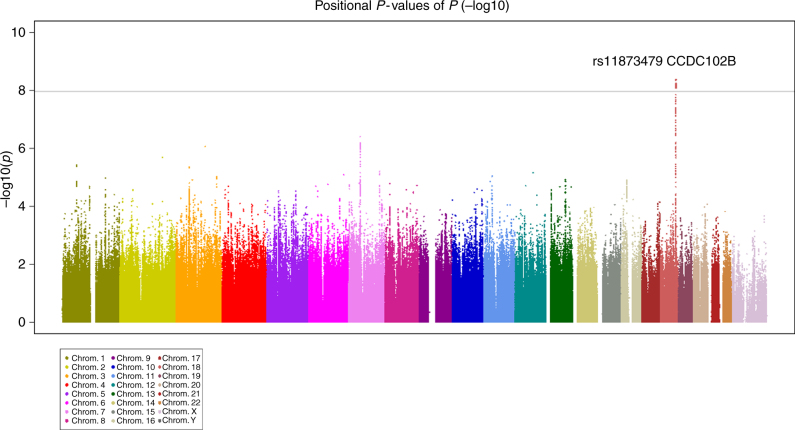
Manhattan plot of case-control GWAS on myopic maculopathy. Each plot shows −log_10_-transformed *P* values for all SNPs adjusted for age, sex, and axial length. The horizontal line represents the genome-wide significance threshold of 1.06 × 10^−8^. GWAS, genome-wide association study

During the first stage with 4462 participants and using a Bonferroni correction threshold of 1.06 × 10^−8^, we identified a genome-wide significant association at *CCDC102B* rs11873439 (*P* = 1.46 × 10^−10^ and *P*_corr_ = 4.26 × 10^−9^, Fig. 1 and Table 1). In the subsequent replication stage with 3279 participants, the *CCDC102B* rs11873439 genotype distribution differed significantly between participants with category 0 or 1 and those with category 2, 3, or 4 (*P* = 4.20 × 10^−4^ and *P*_corr_ = 1.58 × 10^−3^). Meta-analysis of both stages further confirmed the robust association of this locus with myopic maculopathy (*P* = 1.77 × 10^−12^ and *P*_corr_ = 1.61 × 10^−10^).

**Table 1:** Association of rs11873439 (chromosome 18, position 66744288) with myopic maculopathy in the Nagahama cohort

Stage	*N*	Effect allele	EAF		*P*	OR (95% CI)	*N* ^a^	*P* ^a^	OR (95% CI)^a^
			Control	Case					
Discovery	4476	C	0.418	0.529	1.46 × 10^−10^	1.56 (1.36–1.80)	4462	4.26 × 10^−9^	1.63 (1.38–1.92)
Replication	3279	C	0.440	0.505	4.20 × 10^−4^	1.30 (1.12–1.50)	3277	1.58 × 10^−3^	1.30 (1.11–1.53)
Meta-analysis	7755	C	—	—	1.77 × 10^−12^	1.43 (1.30–1.59)	7739	1.61 × 10^−10^	1·46 (1.30–1.64)

EAF effect allele frequency, OR odds ratio, CI confidence interval

^a^ Adjusted for age, sex, and axial length

### *CCDC102B* associates with myopic maculopathy in high myopia

In the discovery and replication stages of the Nagahama GWAS analysis, 828 participants exhibited high myopia with an axial length of more than 26 mm in at least one eye. Within these highly myopic eyes, *CCDC102B* rs11873439 showed significant association with myopic maculopathy (*P* = 2.74 × 10^−4^ and *P*_corr_ = 3.58 × 10^−3^, Table 2).

**Table 2:** Association of rs11873439 with myopic maculopathy in highly myopic eyes

Ethnicity	Stage	Effect allele	Myopic maculopathy (−)		Myopic maculopathy (+)		*P*	OR (95% CI)	*N* ^a^	*P* ^a^	OR (95% CI)^a^
			*N*	EAF	*N*	EAF					
Japanese	Within Nagahama	C	515	0.423	313	0.516	2.74 × 10^−4^	1.45 (1.19–1.78)	828	0.00358	1.43 (1.13–1.82)
	Kyoto, Tokyo	C	190	0.397	968	0.461	0.0221	1.29 (1.03–1.60)	1158	0.0205	1.38 (1.05–1.89)
	Meta	C	705	—	1281	—	2.70 × 10^−5^	1.38 (1.19–1.60)	1986	2.03 × 10^−4^	1.41 (1.18–1.69)
Chinese	SCES	C	134	0.369	41	0.488	0.0458	1.76 (1.01–3.07)	173	0.180	1.64 (0.795–3.40)
Maray	SIMES	C	35	0.386	36	0.444	0.448	1.32 (0.642–2.73)	71	0.713	0.851 (0.360–2.01)
Indian	SINDI	C	62	0.081	23	0.130	0.338	1.68 (0.580–4.88)	85	0.963	1.04 (0.22–4.84)
Meta	5 collections	C	936	—	1381	—	2.40 × 10^−6^	1.40 (1.22–1.61)	2315	1.72 × 10^−4^	1.388 (1.17–1.65)

EAF effect allele frequency, OR odds ratio, CI confidence interval

^a^ Adjusted for age, sex, and axial length

We further conducted replication analysis on the association between *CCDC102B* rs11873439 and myopic maculopathy development using hospital-based high myopia samples (Supplementary Table 2). We collected DNA from 1158 patients with high myopia (axial length > 26 mm in at least one eye) at two facilities in Japan. In this hospital-based analysis of highly myopic eyes, eyes with myopic choroidal neovascularization (mCNV) were also included in the case samples because mCNV usually leads to low vision or blindness. Among this cohort, 190 patients exhibited category 0 or 1 in both eyes, and 968 patients had category 2, 3, or 4 myopic maculopathy or mCNV in at least one eye. The genotype distribution of rs11873439 did not significantly deviate from Hardy–Weinberg equilibrium in either group (*P* > 0.05). This hospital-based analysis revealed the significant association of rs11873439 with myopic maculopathy in high myopia (*P* = 0.0221 and *P*_corr_ = 0.0205), and a meta-analysis of the 828 highly myopic participants in the Nagahama cohort further confirmed its significant association with myopic maculopathy (*P* = 2.70 × 10^−5^ and *P*_corr_ = 2.03 × 10^−4^).

We also performed subgroup analysis of these highly myopic samples categorized by myopic maculopathy grade (Supplementary Table 3). The effect allele frequency in category 2, 3, and 4 samples was higher than that in category 0 and 1 samples; however, the effect allele frequency did not go up with myopic maculopathy grade.

### Replication in other ethnicities and meta-analysis

To confirm the association between *CCDC102B* rs11873439 and myopic maculopathy in highly myopic eyes in other ethnicities, we conducted replication analyses using three different cohorts of three different ethnicities (Table 2). In all three case-control studies using only highly myopic participants, the minor allele frequency was greater in cases, and the odds ratios without adjustment were similar to the Japanese results. The Chinese cohort showed significant association between rs11873439 and myopic maculopathy (*P* = 0.0458) when analyzed without adjustment, although the analysis results substantially varied in the analysis with correction for age, sex, and axial length possibly because of the small sample size. The fixed effect meta-analysis of all ethnicity group data revealed strong association between rs11873439 and myopic maculopathy development (*P* = 2.40 × 10^−6^ and *P*_corr_ = 1.72 × 10^−4^, Table 2).

### Association of *CCDC102B* with axial length

In the entire Nagahama cohort, the association of rs11873439 with axial length was also evaluated because the severity of myopic maculopathy may be associated with longer axial length. Among the 7739 participants, linear regression analysis revealed that rs11873439 showed a mild, but not statistically significant association with axial length (*β* = 0.038 and *P* *=* 0.0782) when adjusted for age and sex.

Subgroup analysis revealed that the odds ratio of the association between *CCDC102B* rs11873439 and myopic maculopathy was similar between axial length of 22–28 mm: 1.51 in eyes with axial length of 22–24 mm, 1.44 in eyes with axial length of 24–26 mm, and 1.45 in eyes with axial length of 26–28 mm (Supplementary Table 4). Although the sample size was small, the effect of *CCDC102B* in the extreme myopia group (axial length > 28 mm) was weaker (*P* = 0.490, odds ratio = 1.29).

### Expression of *CCDC102B* in the human retina and RPE-choroid

**Fig. 2:**
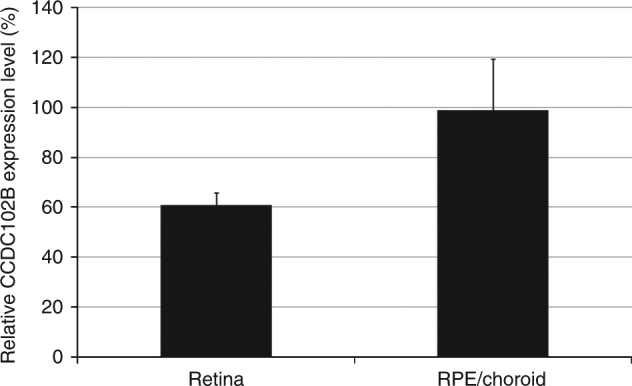
Expression of *CCDC102B* in the human retina and RPE-choroid. *CCDC102B* expression in the human retina and human RPE-choroid normalized to glyceraldehyde-3-phosphate dehydrogenase for cDNA quantification. Human retinal cDNA and RPE-choroid cDNA were obtained from one individual, respectively, and the average of duplicate experiments were used for qPCR analysis. RPE: retinal pigment epithelium. Error bars show standard deviation

To confirm expression of *CCDC102B* in the human retina or RPE-choroid, we conducted SYBR Green quantitative PCR (qPCR) using cDNA from human retina and RPE-choroid. Relative quantification was used to determine the expression of *CCDC102B* with respect to the expression of glyceraldehyde-3-phosphate dehydrogenase (*GAPDH*) as the reference. Stronger *CCDC102B* expression was detected in the RPE-choroid than in the retina (Fig. 2).

### Association of myopic maculopathy with visual disturbance

Visual prognosis was evaluated according to the maculopathy grade in the 818 highly myopic patients from Kyoto University Hospital. Patients were followed up for 51.7 ± 34.9 months, and time course changes in visual acuity were assessed using the Kaplan–Meier method. The eye with the more severe myopic maculopathy grade at the initial visit was selected from right and left eyes for the analysis, and the eye with poorer best corrected visual acuity (BCVA) was used in patients with the same myopic maculopathy grade in both eyes. According to International Statistical Classification of Diseases and Related Health Problems 10th Revision criteria, moderate visual impairment, severe visual impairment, and blindness were defined as 1/10 ≤ BCVA < 3/10, 1/20 ≤ BCVA < 1/10, and BCVA < 1/20, respectively. Low vision included both moderate and severe visual impairment.

**Fig. 3:**
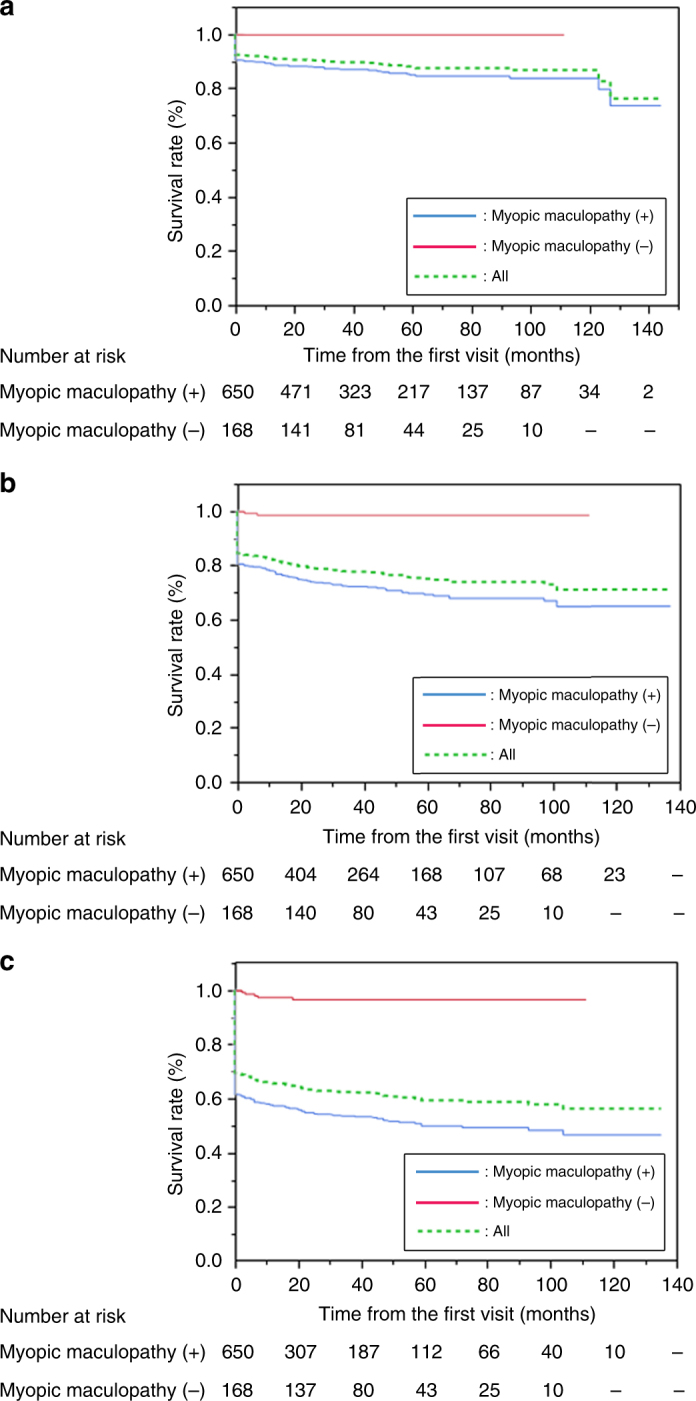
Kaplan–Meier survival analysis for visual acuity decline to low vision and blindness among highly myopic patients according to myopic maculopathy grade. Visual acuity decline to (**a**) 1/20, (**b**) 1/10, and (**c**) 3/10 was observed with a higher frequency in patients with myopic maculopathy grades 2, 3, and 4 at baseline

Figure 3 shows Kaplan–Meier curves of the time to lose 3/10, 1/10, and 1/20 BCVA, with estimated overall survival rates of 57.9%, 73.1%, and 86·8% at 100 months. When survival analysis was also performed for the two subgroups with and without myopic maculopathy, eyes with myopic maculopathy had significantly lower retention rates (48.3% [<3/10], 67.0% [<1/10], and 83.7% [<1/20] at 100 months) compared with eyes without myopic maculopathy (96.8% [<3/10], 98.8% [<1/10], and 100% [<1/20] at 100 months; log-rank test *P* < 0.0001). These results suggested that high myopia accompanying myopic maculopathy was associated with an extremely poor visual prognosis.

## Discussion

Recent increases in the incidence of high myopia are expected to lead to future increases in the rates of low vision and blindness worldwide, although not all eyes with high myopia develop vision-threatening complications. To date, it is not possible to predict which eyes will suffer from vision-threatening complications of myopic maculopathy. Our GWAS using a Japanese cohort showed that *CCDC102B*, encoding coiled-coil domain-containing 102B, located at chromosome 18q22.1-q22.2, had a genome-wide significant association with myopic maculopathy. Furthermore, an analysis of patients with high myopia revealed that the *CCDC102B* locus was strongly associated with the development of myopic maculopathy in highly myopic eyes, and analyses with three ethnicities in Singapore supported this association. These findings, together with the higher rate of low vision and blindness occurrence in eyes with myopic maculopathy observed in the present study, suggested that *CCDC102B* likely determines the fate of highly myopic eyes.

In this study, we provided clear evidence supporting the existence of a pathological difference between the phase of high myopia induction and the phase of myopic maculopathy development. Previous GWASs have discovered genes associated with myopia as well as those associated with high myopia^14–23^. However, *CCDC102B* was not included among the previously reported myopia/high myopia susceptibility genes. Additionally, in our GWAS cohort from Nagahama, *CCDC102B* rs11873439 did not show a significant association with axial length, the most important factor determining the myopic state of an eyeball. The significant association of *CCDC102B* with myopic maculopathy development in highly myopic eyes, even after adjusting for axial length, suggested that *CCDC102B* contributed to the stage of developing myopic maculopathy rather than the stage of inducing high myopia itself. Treatments targeting *CCDC102B* may thus prevent the development of myopic maculopathy and blindness, even after the occurrence of high myopia.

Although the effect of *CCDC102B* on myopic maculopathy was replicated, the effect of *CCDC102B* was clearly attenuated in the replication Japanese cohort as the odds ratio was 1.63 in the discovery cohort albeit 1.30 in the replication cohort. Considering that the odds ratio was 1.43 in the Nagahama cohort myopic samples and 1.38 in the following case-control replication study using hospital-based highly myopic samples, the initial effect size in the discovery GWAS appeared to have been overestimated. In our subgroup analysis, eyes with shorter axial length of 22–24 mm also showed odds ratio of 1.51. *CCDC102B* might affect the development of maculopathy regardless of axial length and there would be a threshold effect going from stage 1 to stage 2, but not beyond stage 2.

In this study, we utilized East Asian populations to find genetic associations with myopic maculopathy development. Although the incidence of high myopia is increasing for all ethnicities, patients with myopic maculopathy are still common in East Asian populations. We found that the C allele frequency of *CCDC102B* rs11873439 in Indians was much lower than that in East Asians. Furthermore, this C allele frequency was reportedly low, ~5%, in Europeans. This result might explain the lower incidence of myopic maculopathy in Caucasians. The best strategy to investigate preventive methods for high myopia-related blindness is thus discovering genes associated with this complication in East Asians, followed by animal experiments to elucidate the mechanisms through which these complications develop; subsequent application of the preventive method to other ethnicities would then be indicated.

Among the participants with high myopia in the Nagahama cohort, 37.8% exhibited stage 2–4 myopic maculopathy. Combined with our longitudinal study results on visual prognosis in highly myopic eyes, 6.0% (0.378 × 0.16) of eyes will suffer from blindness, and 19.7% (0.378 × 0.52) will suffer from low vision within 10 years. As the prevalence of high myopia is now increasing^1–3^, and myopic maculopathy continues to progress in highly myopic eyes^25^, it is anticipated that more cases of low vision and blindness will be encountered in the future.

Although the specific roles of *CCDC102B* have not been reported in any organ to date, one group reported that a 2.7-Mb deletion at chromosome 18q22.1 contributes to congenital diaphragmatic hernia with microphthalmia^26,27^. As this deletion can interrupt *CCDC102B*, *CCDC102B* may affect both microphthalmia and myopic maculopathy development in eyes. In particular, congenital diaphragmatic hernia can develop through damage to the muscle connective tissue, which contains an abundance of collagen and elastin^28^. Notably, collagen and elastin are the main constituents of Bruch’s membrane between the RPE and choroid, where atrophy occurs in myopic maculopathy. *CCDC102B* may thus contribute to the development of diaphragmatic hernia and myopic maculopathy by weakening connective tissue.

Another possibility is that *CCDC102B* may affect actin-myosin cytoskeletal structures. Among the CCDC family, the CCDC88A protein has high homology to *CCDC102B* (BLASTP *E* value = 4.0 × 10^−6^). *CCDC88A*, also termed girdin, has important roles in actin cytoskeleton integrity, retinal angiogenesis, and neurogenesis^29–31^. Moreover, non-muscle myosin II (NMMII) also has high homology to *CCDC102B*; including non-muscle myosin heavy chain IIB (NMMHCIIB, BLASTP *E* value = 4.0 × 10^−9^) and non-muscle myosin heavy chain IIA (NMMHCIIA, BLASTP *E* value = 2.0 × 10^−8^). Both NMMIIA and NMMIIB colocalize with F-actin, and NMMHCIIB depletion reportedly reduces F-actin levels in RPE cells^32,33^. The higher expression of *CCDC102B* in the RPE-choroid as shown by our PCR results supports that *CCDC102B* may promote atrophy of the RPE-choroid by weakening cell integrity and cell adhesion in this tissue through affecting actin-myosin cytoskeletal structures.

In conclusion, we identified a significant association of *CCDC102B* with the development of myopic maculopathy via two-stage GWAS in a community-based cohort and case-control studies using highly myopic patients. Because *CCDC102B* was not significantly associated with the occurrence of myopia, *CCDC102B*-mediated mechanisms to develop myopic maculopathy may constitute promising targets for the prevention of blindness, even after the induction of high myopia. As many eyes with myopic maculopathy will suffer from low vision and blindness in the future, further studies on myopic maculopathy are warranted.

## Methods

### Study participants for the GWAS on myopic maculopathy grade

The study population consisted of healthy Japanese volunteers enrolled in the Nagahama Prospective Cohort for Comprehensive Human Bioscience (the Nagahama Study). Participants were recruited between 2008 and 2010 from the general population of Nagahama City in Japan^16,34^. Community residents ranging from 30 to 74 years of age without physical impairment were eligible for this study. Blood sampling was performed at the time of enrollment. Of the 9804 included participants, 14 withdrew consent to participate and 26 were excluded because genetic analysis showed an ethnic background other than Japanese.

All participants underwent ophthalmic examinations, including an objective determination of the refractive error (Autorefractor ARK-530; Nidek, Gamagori, Aichi, Japan), axial length measurements (IOLMaster; Carl Zeiss Meditec, Inc., Dublin, CA, USA), and color fundus imaging (CR-DG10; Canon, Tokyo, Japan).

Study participants comprised individuals with available DNA samples, fundus imaging, and information regarding age and sex. Other exclusion criteria included the presence of other macular-involving diseases, except for the fundus change owing to myopia, such as age-related macular degeneration, retinal vein occlusion, diabetic retinopathy, or retinitis pigmentosa. Subjects with poor quality fundus images were also excluded.

The Kyoto University Graduate School and Faculty of Medicine Ethics Committee and the Nagahama Municipal Review Board of Personal Information Protection approved the study protocol and procedures used to obtain informed consent. All study procedures were performed according to the tenets of the Declaration of Helsinki. The purpose and procedures of the study were fully disclosed to all participants, and each participant submitted written consent. Analysis was performed after anonymization of patient records and information.

### Genome-wide single-nucleotide polymorphism genotyping

Genomic DNA was prepared from peripheral blood samples according to the manufacturer’s protocol. Samples from 5299 participants who joined the Nagahama cohort from 2008 to 2009 were used for the genome-wide SNP genotyping. The analysis was performed with HumanHap610 Quad (1830 samples), HumanOmni2.5-4 (1616 samples), HumanOmni2.5-8 (378 samples), HumanOmni2.5 s (672 samples), CoreExome24 (1728 samples), and HumanExome (304 samples; Illumina, San Diego, CA, USA).

As a stringent quality control (QC), SNPs with a call rate of less than 99%, minor allele frequency of less than 1%, and significant deviation from Hardy–Weinberg equilibrium (*P* < 1.0 × 10^−6^), and samples with a call rate of <95% were excluded from the analysis. After this QC, 333 participants with estimated first- or second-degree kinship within Nagaham cohort samples (pi-hat > 0.35, PLINK ver. 1.07 [http://zzz.bwh.harvard.edu/plink/]) were also excluded.

Genotype imputation was performed using MACH software (http://www.sph.umich.edu/csg/abecasis/MACH/tour/imputation.html) with the 1000 genomes dataset (phase3 v5 release) as a reference panel. Imputed SNPs for which *R*^2^ was less than 0.5 were excluded from the following association analysis. Finally, 4,710,779 SNPs from 4476 individuals were used for the discovery stage analysis. Of the 4476 samples, 14 lacked data of axial length.

### Grading of myopic maculopathy in the Nagahama cohort

Two ophthalmologists, blinded to participant characteristics, performed grading of the fundus photographs using the International Meta-Analysis for Pathologic Myopia (META-PM) classification system^24^. A senior retinal specialist adjudicated discrepancies. When the grade differed in the right and left eyes, the eye with the more severe META-PM classification was selected for the analysis from each participant.

### Replication for the association study on myopic maculopathy

To conduct a replication study for the genetic association with myopic maculopathy, we genotyped the subset of remaining samples from the Nagahama Study (*n* = 3,421), whose phenotypes were all available, using a commercially available assay (TaqMan SNP assay with the ABI PRISM 7700 system; Applied Biosystems, Foster City, CA, USA). Of these, 69 samples with poor fundus images, 55 samples without fundus images in both eyes, and 18 samples of macular-involving diseases were also excluded. Finally, 3279 samples were used for the replication stage analysis, of which two samples lacked data of axial length.

### Statistical analysis

Genome-wide case-control analysis was conducted for the presence of myopic maculopathy in the discovery set of the Nagahama cohort. Participants with myopic maculopathy grade 2, 3, or 4 in at least one eye were considered cases, and participants with myopic maculopathy grade 0 or 1 in both eyes were considered controls. For every post-QC SNP, we evaluated the association between the genotypes and the presence or absence of pathologic myopic maculopathy using logistic regression, assuming an additive model. This regression framework allowed us to adjust for covariates such as age, sex, and axial length. Experimental-wide significance in the discovery stage was set at 1.06 × 10^−8^, corresponding to *P* = 0.05 divided by the 4,710,779 SNPs included in the analysis. All meta-analyses were performed using a fixed effect model. Differences were considered statistically significant at *P* < 0.05 thereafter. Deviations in genotype distributions from the Hardy–Weinberg equilibrium were assessed using chi-square tests. These statistical analyses were performed using R software (R Foundation for Statistical Computing, Vienna, Austria; available at http://www.rproject.org/), JMP^®^ 12 (SAS Institute Inc., Cary, NC, USA), and PLINK software.

### Association with myopic maculopathy in eyes with high myopia

Japanese patients with high myopia were recruited from the Kyoto University Hospital (*N* = 818) and Tokyo Medical and Dental University Hospital (*N* = 340), excluding patients with other ocular diseases such as retinal vein occlusion, diabetic retinopathy, severe glaucoma, or retinitis pigmentosa. High myopia was defined as axial length more than 26 mm in at least one eye. All procedures were performed according to the tenets of the Declaration of Helsinki after approval of the protocols by the Institutional Review Board and the Ethics Committee of each participating institute. The purpose and procedures of the study were fully disclosed to all participants, and each patient submitted written consent.

Grading of the fundus photographs in both eyes was performed using the META-PM classification^24^. Genotypes of the 818 patients admitted at Kyoto University Hospital were determined using TaqMan allelic discrimination probes (Applied Biosystems). A series of BeadChip DNA arrays, namely HumanHap550 Quad (195 samples) and Human660W-Quad BeadChip (145 samples), were used to determine the genotypes of patients in the Tokyo Medical and Dental University Hospital, with the genotype of rs11873439 being directly determined by the chip. We assessed long-term visual impairment from myopic maculopathy by Kaplan–Meier estimates using the 818 patients with high myopia from Kyoto University Hospital.

### Association of *CCDC102B* in high myopia in other ethnicities

Participants with high myopia were selected from the Singapore Chinese Eye Study (SCES), Singapore Malay Eye Study (SiMES), and Singapore Indian Eye Study (SINDI); the samples included 175 Chinese samples, 71 Malaysian samples, and 85 Indian samples^35,36^. High myopia was defined as SE of −5.0 D or less for the right eye, and grading of the fundus photographs of right eye was performed using the META-PM classification. Age, sex, axial length in right eye, and principal components were used as covariates in the logistic regression analysis. In the SCES cohort, HumanHap610 Quad (132 samples) and Omniexpress chip (43 samples) were used to determine the genotypes of rs11873439, and both the data were subjected to meta-analysis. In both SiMES and SINDI cohorts, samples were genotyped using the Illumina Human610 Quad Beadchip.

### Quantitative real-time polymerase chain reaction

Human retinal cDNA and RPE-choroid cDNA were obtained from ScienCell Research Laboratories (Carlsbad, CA, USA) and BioChain Institute, Inc. (Newark, CA, USA). The SYBR Green qPCR reaction mixture contained cDNA, forward primer, reverse primer, ROX reference dye, and SYBR premix ExTaq^TM^ II (TaKaRa Bio, Osaka, Japan). The reactions in 96-well plates were performed using the Stratagene MX3000P real-time PCR system (Santa Clara, CA, USA). The thermal cycle conditions were as follows: 30 s at 95 °C, followed by 40 cycles at 95 °C for 5 s and 60 °C for 31 s. Relative quantification was used to determine the expression of *CCDC102B*. The expression of *GAPDH* was used as the reference in the qPCR analyses.

The primer sequences were 5′-CTCTGCTCCTCCTGTTCGAC-3′ (forward) and 5′-GCCCAATACGACCAAATCC-3′ (reverse) for *GAPDH* and 5′-TCATGGGCTACAATCTCATGCT-3′ (forward) and 5′-CGCAGGCGAAGTTCTTCAC-3′ (reverse) for *CCDC102B*. All reactions were performed in duplicate, and the average values of experiments were used for analysis.

### Data availability

The data that support the findings of this study are available from the corresponding author upon reasonable request.

## Electronic supplementary material

Supplementary Information
