# Supplementary material for: CCDC102B confers risk of low vision and blindness in high myopia
Source: Nat Commun. 2018 May 3;9:1782. doi: 10.1038/s41467-018-03649-3 (PMC5934384; doi:10.1038/s41467-018-03649-3)

CCDC102B confers risk of low vision and blindness in high myopia

Hosoda et al.

Supplementary Table 1. Description of the Nagahama cohort for our two-stage genome-wide association study on myopic maculopathy.

Stage	N	Female (%)	Age (years)	Myopic maculopathy grade					AL (mm)
				0	1	2	3	4	
Discovery	4462	3014 (67.5%)	52.0 ± 13.5	3640	371	427	20	4	24.19 ± 1.43
Replication	3277	2231 (68.1%)	54.4 ± 12.4	2563	300	393	17	4	24.11 ± 1.44

AL, axial length

Supplementary Table 2: Characteristics of participants in the high myopia study.

	**Nagahama**	**Kyoto**	**Tokyo**	**SCES**	**SIMES**	**SINDI**
N	828	818	340	175	71	85
Female (%)	506 (61.1%)	492 (60.1%)	224 (65.9%)	91 (52.0%)	36 (50.7%)	23 (27.1%)
Age (years)	47.6 ± 12.7	61.4 ± 13.6	55.2 ± 13.3	53.4 ± 7.1	54.7 ± 11.1	45.9 ± 11.3
Myopic maculopathy grade						
0	371	16	3	83	10	19
1	144	154	22	51	25	43
2	275	318	91	30	32	23
3	31	144	92	11	4	0
4	7	186	132	0	0	0
mCNV (+)	0	225	150	0	0	0
AL (mm)	27.05 ± 1.06	28.75 ± 2.00	30.38 ± 1.53	26.60 ± 1.40	26.65 ± 1.60	26.03 ± 1.57

mCNV, myopic choroidal neovascularization; AL, axial length

Supplementary Table 3: ***CCDC102B*** **rs11873439 effect allele frequency in Japanese population with high myopia.**

**Myopic maculopathy grade**		**0**		**1**		**2**		**3**		**4**		**Total**	
	**Effect allele**	**N**	**EAF**	**N**	**EAF**	**N**	**EAF**	**N**	**EAF**	**N**	**EAF**	**N**	**EAF**
Nagahama	C	371	0.427	144	0.413	275	0.522	31	0.516	7	0.286	828	0.458
Kyoto, Tokyo	C	18	0.417	172	0.395	335	0.452	151	0.447	482	0.472	1158	0.451
Total	C	389	0.427	316	0.403	610	0.484	182	0.459	489	0.469	1986	0.454

EAF, effect allele frequency.

Supplementary Table 4: Association of rs11873439 with myopic maculopathy in the subgroups categorized with axial length using Nagahama cohort samples.

Axial length (mm)	Effect allele	Myopic maculopathy (-)		Myopic maculopathy (+)		P	OR (95% CI)	P*	OR (95% CI)*
		N	EAF	N	EAF				
< 22	C	211	0.455	4	0.625	0.339	2.00 (0.47 – 8.46)	0.313	2.12 (0.49 – 9.05)
22-24	C	3643	0.425	223	0.514	2.52×10^{-4}	1.43 (1.18 – 1.73)	4.67×10^{-5}	1.51 (1.24 – 4.07)
24-26	C	2505	0.427	325	0.520	7.14×10^{-6}	1.45 (1.23 – 1.71)	4.37×10^{-5}	1.44 (1.21 – 1.72)
26-28	C	491	0.422	222	0.523	3.92×10^{-4}	1.50 (1.20 – 1.88)	0.00479	1.45 (1.12 – 1.87)
28 <	C	24	0.458	91	0.500	0.608	1.18 (0.62 – 2.24)	0.490	1.29 (0.63 – 2.64)

EAF, effect allele frequency; OR, odds ratio, CI, confidence interval.

*adjusted for age, sex, and axial length

Supplementary Figure 1: Quantile-quantile (QQ) plots from the discovery stage.

The QQ plot for associations between all analyzed single nucleotide polymorphisms (SNPs) and myopic maculopathy in the discovery stage. Each blue dot represents an observed statistic (defined as $-\log_{10}$ P) versus the corresponding expected statistic before genomic control, while each red dot represents the observed statistic versus the corresponding expected statistic after genomic control. The black line corresponds to the null distribution.

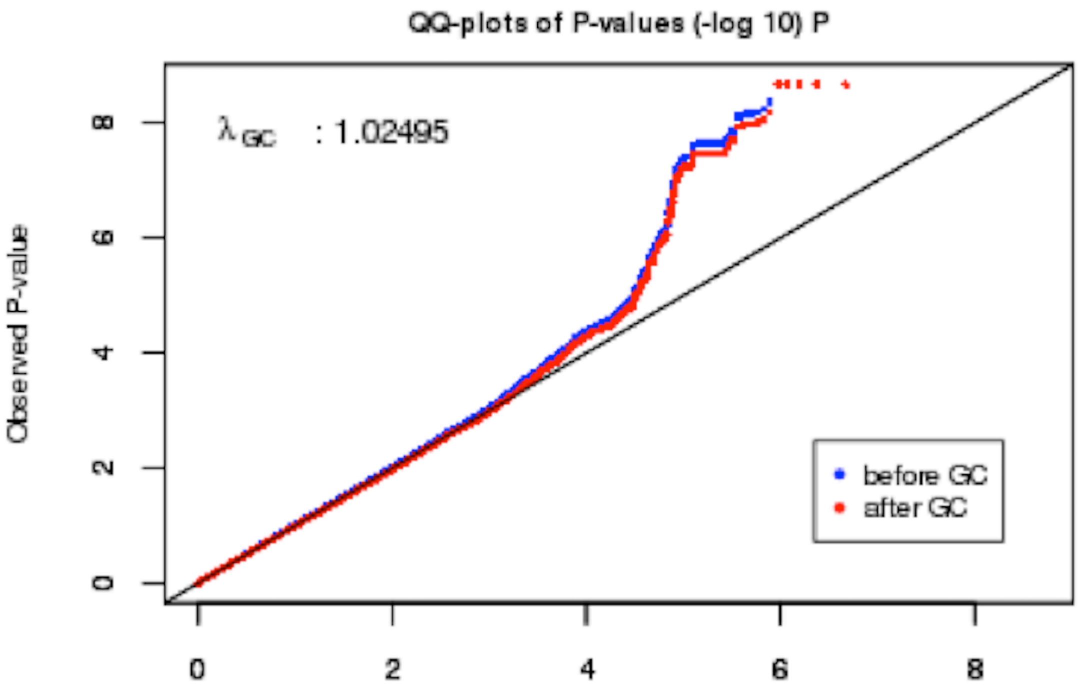

Supplement: Supplementary file 1 — Supplementary Information [file 41467_2018_3649_MOESM1_ESM.pdf]
